# Ten Simple Rules for Curating and Facilitating Small Workshops

**DOI:** 10.1371/journal.pcbi.1004745

**Published:** 2016-07-21

**Authors:** Greg J. McInerny

**Affiliations:** 1 Centre for Interdisciplinary Methodologies, Department of Social Sciences, University of Warwick, Coventry, United Kingdom; 2 Department of Computer Science, University of Oxford, Oxford, United Kingdom; Dassault Systemes BIOVIA, UNITED STATES

As a participant, workshops are by far my favorite scientific event. Compared to conferences, the interactions can be more intense, discussions can be deeper, and the resulting collaborations are often stronger. Working with 10–30 attendees over a few days can lead to a more open and integrated event than a conference. At workshops, you are a participant in the whole event, and you can make many direct contributions to its goals. In contrast, at conferences, the aim is for a broad informational in which you are part of the audience and contribute comparatively little content.

As an organizer, workshops will present you with diverse challenges. You will manage the project and its logistics [1], and you will also be the curator(s) by developing and negotiating workshop content. Possibly the least well recognized role is as facilitator(s), when you enable interactions amongst participants and workshop activities. In addition to organizational skills [1], a workshop will profit from your creativity, empathy, and mediation skills. These ten simple rules make links between these roles and aim to help you reach your goals whilst making an enduring contribution to your community [2].

There is no single formula for creating good workshops. In contrast to the fairly standard format of conferences (plenary, coffee, talks, lunch, talks, coffee, talks—repeat), workshops can take diverse forms, and indeed, they should to fit different goals. For example, different workshops are needed when exploring a single research topic, initiating a working group, developing interdisciplinary collaborations, or testing new methods and software (e.g., compare [1] to [3]). Different workshop goals will then require different kinds of attendees, timetables, interactions, props, atmospheres, etc. The details of your roles as organizer(s), curator(s), and facilitator(s) will also differ between different types of workshops and will develop as your experience and confidence grows.

Developing workshops can involve jeopardy. The organization, curation, and facilitation can, in places, go wrong. Participants’ time away from their work and personal lives should be worthwhile (also see [4]). Tangible outputs can also be hard to develop in a short period and may not always involve you. Most obviously, the workshop will divert time you would spend on research and other parts of your job. It is perhaps the wrong framing to see workshop organization as a “time- and energy-draining black hole” [5]. Like all worthwhile things, workshops will require your time and energy, but that needn’t be draining. Instead, you can make it a rewarding and energizing experience. Your dedication and enthusiasm will reduce the jeopardy and increase the productivity of the workshop. You will find greater enjoyment in the whole process, too.

## Rule 1: Assess Past Successes and Failures

Some workshops are better than others. But why? Was it the organizers, attendees (see Rule 3), goals, size, subject matter, timetabling, activities, venue, location, seating plan, seats, timetable, time keeping, props, access to plug sockets, projection equipment, biscuits, heating, travel, weather? Individually, any of these can be a minor factor. Some, like appropriate attendees and clear goals, will directly influence the success or failure of your workshop. Take control of what you can, and make the event what you want it to be. Think big and small.

Make workshops part of your conversations. Find out what people like or have found irritating. Explore your contacts for organizers of workshops and events. Find out what succeeded and why. You might ask them to be a co-organizer. Workshops should vary, so don’t follow someone else’s template uncritically. Most of all though, be clear about what you want to achieve. Is a workshop really the best process to achieve that goal?

## Rule 2: Develop a Brand

**Fig 1:**
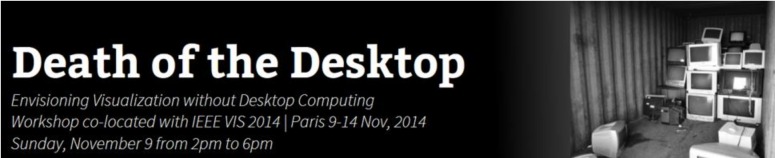
Branding needn’t be demanding. The “Death of the Desktop” workshop (see: http://beyond.wallviz.dk/, https://vimeo.com/102527731) had a strong brand that was intriguing, evocative, and memorable. The twitter account (@visfutures) revealed information and contributions in the lead-up to the event. The brand helped to set a tone for the preworkshop submissions and the workshop activities. Consider how effective the brand would have been if the workshop was just called “Consequences of a concerted community focus on visualization technologies and the impact on interaction and display techniques.” Would this have been easy to remember or Google? Would this have set a theme that was easy to follow?

Branding helps you engage with people throughout the development, execution, and reporting of your workshop. A brand should efficiently explain what you will do and why (e.g., a catchy workshop name, a recognizable logo, and a tweetable mission statement or statement of goals). Successful brands needn’t require huge investments of time, and brands needn’t be loud to be noticed. Aim for “salient” rather than “extravagant” (Fig 1).

Ignoring branding increases your chances of miscommunications and missed opportunities. Your brand will support you with: (a) pitch (securing funding, inviting participants, informing speakers and co-organizers), (b) function (developing identity and focus, producing printed materials and signage, a succinct reminder of the workshop goals), (c) recognition (reporting, gaining attention and reputation, developing buy-in and helping people find the website), and (d) decision making (a frame of reference for both organizers and participants). Brands are just as important, if not more, for single workshop events where outcomes (however small) must be produced in a short time. Also, your invitations may have a less substantial reputation to rely on and must stand out amongst busy email inboxes.

Importantly, you will be the most visible and vocal component of your brand, the one thing that interfaces with everything else. Be prepared to put yourself out there and represent and bring coherence to the workshop.

## Rule 3: Recognize Diversity and Use It

Workshops become conferences if they are too big, have too many talks, or if you invite an audience rather than participants. Workshops become group meetings if too many people attend from one organization or project, become training events if there are too many early-career researchers, and become board meetings if there are too few. Boring workshops emerge from too much agreement, too little participation, or too little openness. Workshops depend on diversity, so be prepared to be purposeful with inviting participants, and don’t be afraid to engage in a bit of social engineering (also see [3]). Some workshop dynamics can be hard to foresee, but others are very predictable.

Invite a diversity of characters, skills, and knowledge that will make your workshop work. Invite provocative characters that can ignite discussions or will be likely to play devil’s advocate, and invite those who can provide syntheses to multifaceted discussions. Invite potential authors for paper outputs. Why not invite the editor of a journal? The right attendees are fundamental to achieving your goals.

Group activities require rapporteurs, facilitators, and contributors as well as skills such as drawing. If groups self-select and deliberate on task management, you will waste time, de-energize participants, and disrupt the flow. So, you can assign groups (and seating plans, Rule 6) with purpose. These groupings should ensure function. For instance, if vocal individuals will dominate discussions, then place early-career researchers or quieter individuals in their own group. Use specialist groups to hear discipline-specific views, and use multidisciplinary groups to understand how to integrate different perspectives. Create structure from the diversity.

## Rule 4: Tell Speakers What to Say

Organizers, participants, and speakers all appreciate when talks are relevant, so offer appropriate direction so that presentations fit the workshop goals. This will require some negotiation and gentle editorial input. If you want a particular presentation, you will have to ask! Without direction, speakers can unintentionally divert a workshop’s path. Provide context on the goals and the backgrounds of participants beforehand. Some speakers really enjoy new challenges, so you might ask for something nonstandard; for example, a view from the shop floor or an outsider’s view on a topic. It may just change the wording of an existing talk rather than the whole presentation. Either way, all speakers like to be relevant and on point.

Not everyone will adjust their talk based on overlap with preceding talks. So, where possible, reduce the potential for awkwardness and repetition by breaking down topics into modules. You might encourage speakers to consult with each other, or consider sharing your introduction so they can edit overlapping material. This can help direct your own presentation (also see [6]).

## Rule 5: Learn to Love Emailing

Emails are the backbone of workshop preparations (invites, replies, accommodation, replies, requests, replies, travel plans, replies…phew!). It can be tempting to hand off many of these tasks (hundreds of emails for a workshop? It has been known!). However, emails are opportunities to build relationships and uncover opportunities. You may have the support of event organizers, but work with them rather than being dependent on them.

Emails don’t have to be too formal and should encourage a dialogue. Emails can help you understand participants’ backgrounds, motivations, and expectations that can then reveal any concerns and suggestions they may have and may also help you consider seating arrangements (Rule 6). Not every email will be interesting. Most won’t. Taking responsibility means information is not lost on the way to someone else’s spreadsheet. You can tailor invitations to different kinds of people and different disciplines (“you would really find X, Y and Z of interest”), offer help before being asked (e.g., travelling with a partner = double room, or being family-friendly [7]), address concerns at an early stage (“thanks for the invite but I don’t think I am the right person*…*”), and pick up opportunities (“our organization would like to sponsor the workshop”).

Whilst many tools can make mundane tasks easy (e.g., SurveyMonkey, Doodle, Google Forms), consider when group requests for information are suitable or when individual emails may be better. A poorly pitched group email can spark off many individual responses. Be prompt in your own replies, as participants and event administrators will need information at certain times.

## Rule 6: Seriously, Consider Your Seating Plan Seriously!

Conference-style, row seating plans can stifle productive workshop dynamics. A coat laid on a seat, people occupying row ends, and a lack of eye contact can all inhibit important introductions and exchanges. Seating plans should account for practical matters (view of projector screens, fire exits, position of plug sockets, or access to workshop materials and props) and support the flow of your timetable and the interactions amongst participants. It is actually a big deal, so take it seriously! Also, see [3].

**Fig 2:**
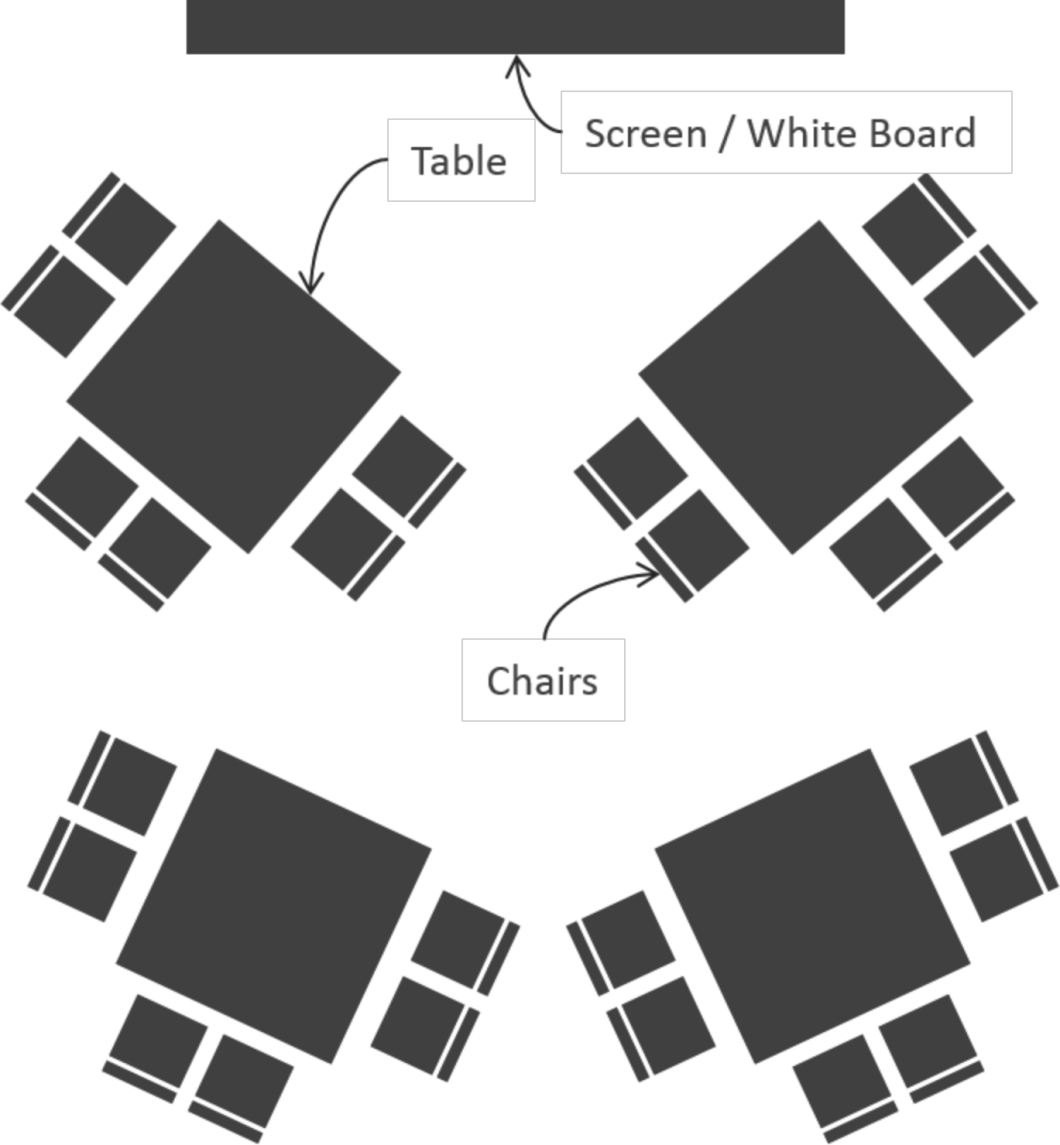
Cabaret seating plans (also see [3]) can support different workshop activities. A seating plan may help you distribute facilitators and rapporteurs (See Rule 3).

“Cabaret” seating plans can help workshops to function (Fig 2). People can maintain views of screens and speakers whilst allowing face-to-face contact in discussions. In addition, switching between whole and smaller group activities is made easy. A named seating plan can reduce uneasiness and help direct different groupings for different activities or days (if you know people and their work sufficiently well). A spare chair or two at the back may allow people to find a bit of thinking space. Think ahead and don’t be afraid to ask venues for what you want.

## Rule 7: Document the Workshop Using Fresh Resources

There are many opportunities to go further than traditional recordings or note taking. Twitter can help you in a variety of ways [8], including recording key points, links, names, and organizations in a format that is immediately shareable. You can also engage audiences beyond the workshop and solicit their views or questions. Active tweeters make this easier, so invite some! Leave some tweets before the event as breadcrumbs and also tweet a link to the workshop website with an event hashtag. However, tweets may not always be essential or appropriate to the workshop material and sometimes can even be a distraction.

Figshare, SlideShare, and related repositories make it easy to disseminate and discover talks if they are tagged appropriately. Tweeting links to talks can generate extra interest and value for your participants. Relevant or selected tweets and documents can also be recorded (e.g., storify.com) or retweeted via a workshop twitter account. Not everything has to be shared though, and you might consider a charter that guides the dissemination of material beyond the workshop.

**Fig 3:**
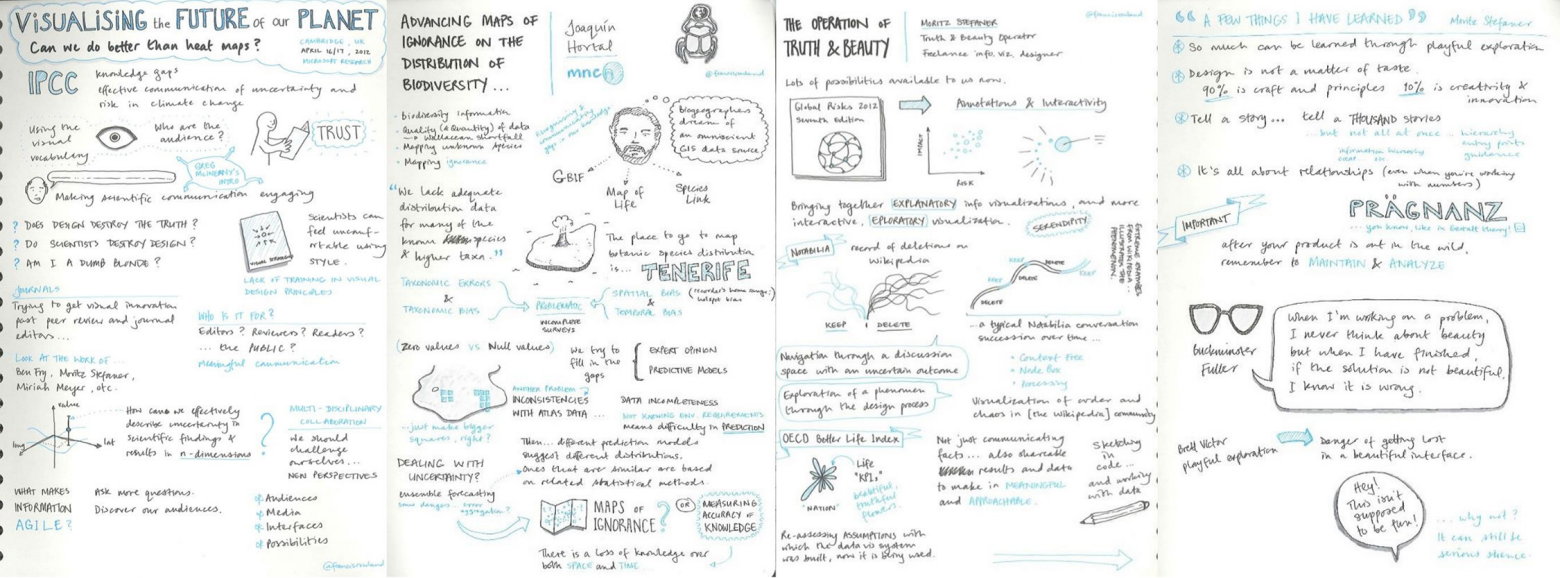
Sketchnotes (http://www.flickr.com/photos/francisrowland/6944419112/lightbox/ | @francisrowland).

At one workshop, a very talented participant showed me their sketchnotes (Fig 3)—“purposeful doodling” (http://sketchnotearmy.com/about/)—that record selected content from talks or discussions. Sketchnotes can be more evocative and memorable, capturing key points or images or adding context by drawing themes together. Find a sketchnoter and invite them along. Brief them on the workshop beforehand.

## Rule 8: Include Something Unexpected

Executing a standard workshop format is demanding and worth trying in the first place [9]. When your confidence has grown, you may consider more inventive activities that break up routine interactions and allow you to go in different directions. This could be simple, from inviting an unexpected speaker to a more involved approach with structured activities, such as “gamestorming” (http://www.gogamestorm.com/).

Used critically, gamestorming can help you dissect, characterize, and explore ideas or problems in ways that discussions cannot. By harnessing participants’ creativity and skills other than talking from a crowd, these “games” can produce much more inspired responses to problems by breaking down preconceived ideas and building new perspectives. Participants can find flaws in these simple games, but that is often useful in itself. It can also be “fun.”

Unexpected speakers can help explore the extremes of topics. It’s a simple and effective strategy. They may be people who use different tools for similar research, similar tools for different research, or are just somehow unusual in their field. Deliberately stretching a topic’s boundaries can stretch the workshop’s possibilities. At a scientific workshop, invite an information visualization speaker; at a visualization workshop, invite a social scientist; and at a multidisciplinary workshop, invite a communications expert. Your job is to ensure that this strategy brings value and isn’t just a diversion.

Remember that there aren’t really any rules. Presentations don’t have to have equal time. If topics are familiar, then allocate less time; if they’re unfamiliar, add more time for both presentation and questions. You can pair talks and leave questions for a discussion afterwards, use the popular “ignite talks” formula, have a demo session over coffee, use Pecha Kucha (http://en.wikipedia.org/wiki/PechaKucha), incorporate an “unconference” component in which some activities are crowdsourced [10], or go for a hike together. Or do all of those. Be inventive. You don’t have to do what everyone else has done before. Overly complicated timetables can, however, become bewildering, and hikes may require wellies, so be sensitive to your participants.

## Rule 9: Prepare for the End

Final impressions are important. How you close a workshop can affect how participants perceive a workshop’s success and how they judge their own contributions. So, invest in the workshop’s closing [1]. It should match the vibrancy of your event. If you finish early, what does that say to the participants?

Prepare a structure for your final words and any thank-yous. The gaps can be filled in during the workshop; e.g., record take-home points from talks and activities, key suggestions, solutions, or any extra thanks that should be noted. Do not make too many unplanned promises though! Many workshop papers are never written. Gauge support and seek informal feedback before launching into public requests for significant work after the event. This avoids the tumbleweed (silence) when asking who would like to lead on a particular output.

At the end, you and the participants will be at your most exhausted and gorged on information and starting to think of return journeys, work, and families. Asking participants for a take-home message can be awkward and ineffective at this point. Even with only 20 or so people, tired, on-the-spot reflections can provide a damp ending. You can brief and plant selected rapporteurs beforehand if needs be (use diversity strategically, Rule 3).

## Rule 10: Don’t Panic, Things Go Wrong

As with the rest of life, things can go wrong. It’s how you respond that matters. Don’t panic, and deal with problems with a smile. Many of these simple rules have emerged from three types of problems.

First, problems with organization and planning: food may not arrive, dietary requirements may not have been passed on, and speakers may forget your previous discussions (Rule 4). A venue might inform you that it has removed projection equipment, Wi-Fi, flipcharts, furniture, and all facilities support a week before (it has happened!). Do not look for fault or blame. Just focus on solutions, and act quickly. Double check details close to the actual date. If possible, visit venues beforehand and check seating or even taste food if that is possible. Not all spring rolls are created equal.

Second, problems with not sticking to your plan: unexpected discussions or opportunities can tempt you to deviate from your timetable. Groups may not have completed a task on time. Or, you may panic about an activity’s relevance and ditch it. Improvising can trip up the workshop flow or put pressure on people and activities later in the schedule, so be very sure that the alternative is better and be decisive. You don’t have to accommodate on-the-spot requests to change the timetable or talk about a particular subject, even if the request comes from someone more senior. Sticking to a well-thought-out plan is often the best response. A good discussion will find its own way of continuing, so don’t worry about exhaustively working through every item to a conclusion.

Thirdly, unexpected problems: some things just happen and you can’t do anything about it. Don’t panic. Do what you can and then go with the flow. The worst cases are illness during workshops and mislaid plane tickets. You can’t do much except making sure the participants’ needs come before those of the workshop. It is usually a good story after the event.

## Summary

Rather than overcomplicating your workshop plans, you need to strike a balance amongst what things would be interesting to do and those that have to be done. It can be easy to overload yourself and for your enthusiasm to turn into a tendency to be overzealous. Achieving a balance may also require that you apply these rules with an appropriate pinch of salt. For example, you may feel branding (Rule 2) is superfluous, recording all talks via social media (Rule 7) may not suit everyone, and you may want to encourage unexpected material (Rule 8) by not telling speakers what to say (Rule 4).

However—and this is really the aim of all the rules—in order to make those decisions, you need to be reasonably well informed about what workshop organization will and could involve. The requirements and possibilities aren’t always obvious and are broader than these ten simple rules (see [1], [3], [7], and [9]). Even if you go for the “simple” option, it is worth being aware of what opportunities or problems you are excluding or encouraging; for instance, if you were to ignore the benefits of social engineering (Rule 3), seating (Rule 6), or communications (Rule 5) or by being unprepared for timetable changes (Rule 10) and the workshop closing (Rule 9). Good luck! I have never got everything right at the same time!
